# Proximal 4p deletion syndrome in a woman with intellectual disability: a case report and literature review

**DOI:** 10.1186/s13039-025-00735-2

**Published:** 2025-11-03

**Authors:** Liqiang Wei, Yu He, Denghe Liu, Xiaojv Chi, Xi Qin

**Affiliations:** 1 Department of Clinical Laboratory, the First Affiliated Hospital of Guangxi Medical University, Nanning, 530000 Guangxi Province China; 2 Key Laboratory of Clinical Laboratory, Education Department of Guangxi Zhuang Autonomous Region, Medicine of Guangxi Medical University, Nanning, 530000 Guangxi Province China; 3 Department of Clinical Laboratory, Baise Maternal And Child Hospita, Baise City, 533000 Guangxi Province China

**Keywords:** 4p15.32-16.2, Interstitial deletion, 16p13.13 duplication, Intellectual disability, Ovarian dysfunction

## Abstract

Deletions in chromosome 4p can lead to two distinct phenotypes, Wolf-Hirschhorn syndrome (WHS) and proximal 4p deletion syndrome. While WHS, associated with distal deletions, has well-characterized phenotypic features, proximal 4p deletion syndrome, involving the 4p14-p 16.1 region, shows moderate manifestations, and its causative gene remains unknown with fewer reported cases. Here we report a Chinese case: a 21-year-old female with a peripheral blood chromosomal karyotype of 46,XX, del(4)(p15.3-p16). NGS-CNVA further revealed an 11.7 Mb deletion in the 4p16.2-p15.32 region and a 1.25 Mb microduplication in 16p13.13. She had ovarian dysfunction, and moderate Intellectual Disability(ID) without typical proximal 4p deletion phenotypes. Through analysis of Genecards and OMIM databases, we identified two neurodevelopmental genes *DRD5* and *WFS1*, and four ovarian dysfunction-related genes *WFS1*,* CC2D2A*,* PROM1*, and *QDPR*, suggesting their roles in the patient’s manifestations. Additionally, a review of 37 published cases of proximal 4p deletion syndrome revealed 17 cases with an overlap in the deleted region with our case. This report not only enhances the recognition of this rare syndrome among clinicians but also provides a basis for further exploration of the potential causative genes, contributing to a better understanding of the genotype-phenotype correlations in proximal 4p deletion syndrome.

**Supplementary Information:**

The online version contains supplementary material available at 10.1186/s13039-025-00735-2.

## Introduction

Chromosomal deletions involving the short arm of chromosome 4 (4p) have been associated with two distinct clinical phenotypes: Wolf-Hirschhorn syndrome (WHS) and proximal 4p deletion syndrome. WHS, primarily caused by distal deletions in the 4p16.3-pter region, is characterized by distinctive craniofacial dysmorphology (e.g., trigonocephaly, broad nasal bridge, and hypoplastic nasal alae), severe developmental delay, and intellectual disability [1, 2]. In contrast, proximal 4p deletion syndrome, defined by deletions in the 4p14-p16.1 region, presents with moderate clinical manifestations, including mild to moderate intellectual disability, subtle dysmorphic features (e.g., long face, up-slanting palpebral fissures, epicanthal folds, and large lips), and skeletal abnormalities (e.g., pectus excavatum, genuvalga, and joint hyperlaxity) [3, 4].

Despite increasing reports of proximal 4p deletions, the syndrome remains understudied, with approximately 37 cases documented in the literature [5]. Most prior studies relied on conventional karyotyping, limiting the precision of deletion mapping; only a subset of cases have been characterized using high-resolution genomic techniques [6–9]. The molecular basis of proximal 4p deletion syndrome remains unclear, as causative genes have not been definitively identified. Notably, phenotypic variability among reported cases further complicates research. While some patients exhibit classic features like developmental delay and dysmorphology, others present with atypical manifestations [10, 11].

Here, we described a Chinese case of proximal 4p deletion syndrome, characterized by an 11.7 Mb deletion in 4p15.32-p16.2 and a 1.25 Mb microduplication in 16p13.13. The patient presented with ovarian dysfunction, and moderate intellectual disability(ID), lacking typical craniofacial features of proximal 4p deletion syndrome. Through genomic database analysis (Genecards and OMIM), we identified candidate genes (e.g., *WFS1*,* DRD5*,* CC2D2A*,* PROM1 and QDPR*) that may contribute to her phenotypes. Furthermore, a systematic review of 37 published cases revealed 20 with overlapping deletion regions, enabling comparative analysis of genotype-phenotype associations. This report aims to: (1) expand the clinical and genetic spectrum of proximal 4p deletion syndrome, (2) provide new insights into potential causative genes, advancing our understanding of this rare syndrome. We hope this report will help make this syndrome more recognizable among clinicians and provide a foundation for identifying the primary causative gene.

## Patient presentation

The proband was a 21-year-old female, who presented to our outpatient service due to primary infertility and ovarian dysfunction. Intellectual Disability Level 3 based on the assessment results of the World Health Organization Disability Assessment Schedule 2.0 (WHODAS 2.0). She was able to perform basic activities of daily living, including personal hygiene, and feeding herself. Her medical history revealed that she attained menarche at the age of 13 and had regular menstrual cycles.

On physical examination, the patient exhibited normal vital signs and systemic functions. Her external genitalia appeared normal, and she had a normal-sized cervix. Transthoracic echocardiography revealed no abnormalities. Laboratory investigations, including routine blood parameters, liver and kidney function tests, myocardial enzymes, blood coagulation profile, thyroid hormones (TH), human growth hormone (hGH), adrenocorticotropic hormone (ACTH), and sex hormones, were within normal limits. There was no history of seizures according to her parents. The patient presented with a tall and thin body habitus but lacked minor dysmorphic features associated with proximal 4p deletion syndrome. The patient did not undergo specific therapeutic interventions related to her chromosomal abnormality. However, she was counseled regarding the potential implications of her condition on fertility and reproductive health. The proband also had a sister and a brother, who were healthy, but their karyotypes were unavailable. The patient’s parents were healthy and did not undergo routine chromosomal analysis.

## Results

**Fig. 1:**
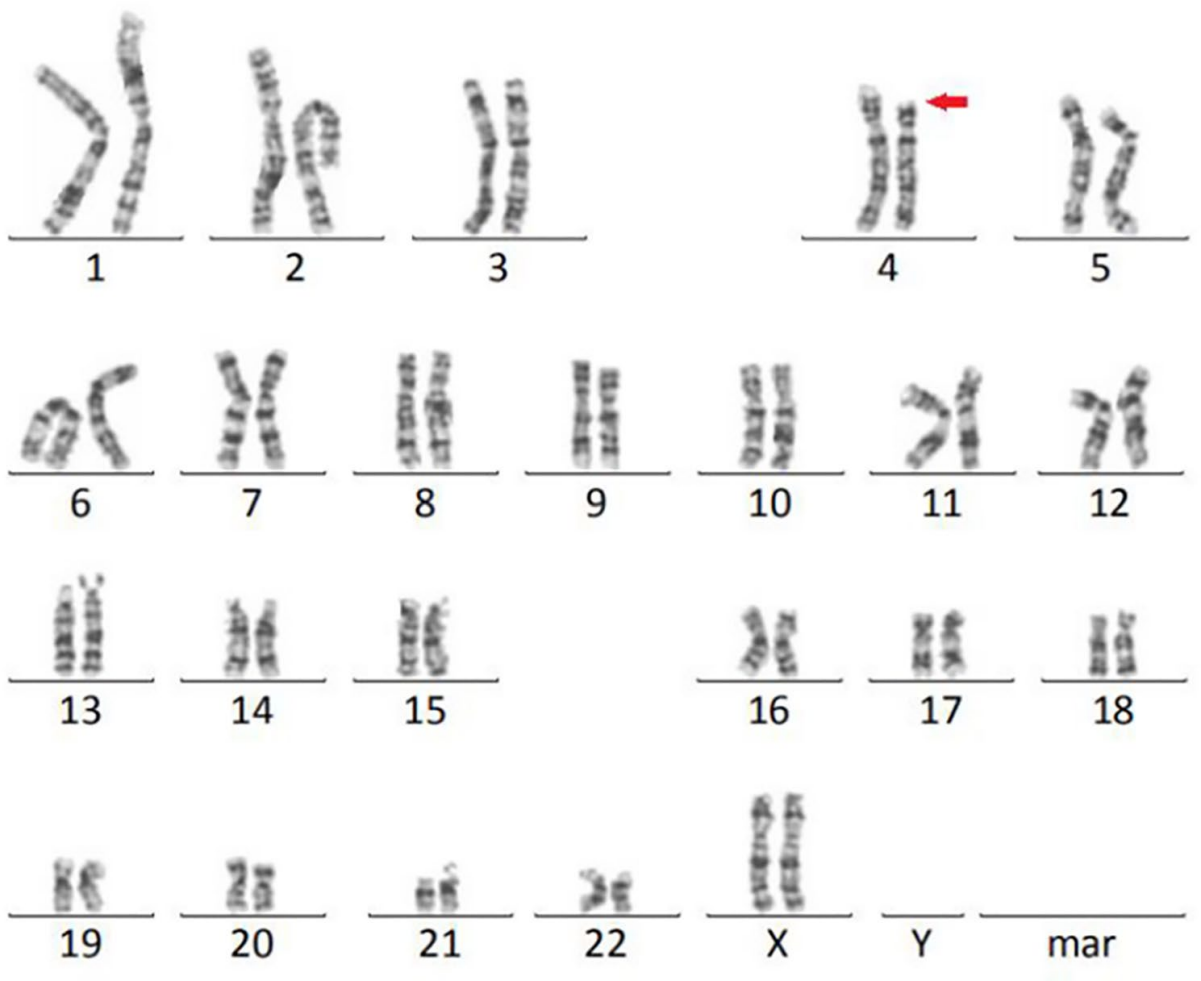
An interstitial deletion in 4p was revealed by conventional G-banding chromosome analysis

**Fig. 2:**
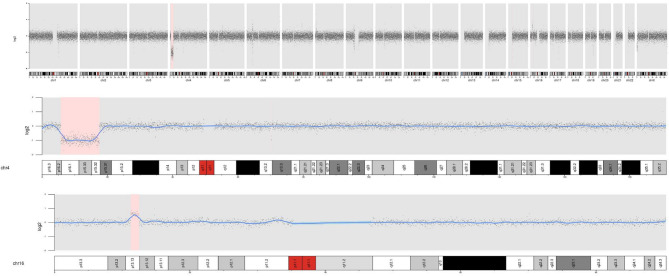
NGS-CNVA results showing one copy of deletion on 4p15.32-p16.2 and one copy of duplication on 16p13.13

Routine chromosomal analysis using standard G banding revealed a deletion of the short arm of chromosome 4, designated as 46, XX, del(4)(p15.3-p16) (Fig. 1). Subsequent NGS-CNVA testing on the patient’s genomic DNA showed an approximately 11.7 Mb deletion in the 4p15.32 - p16.2 region: seq[GRCh38] del(4)(p15.32p16.2) chr4:g.5776037_17548280del and an 1.25 Mb duplication in the 16p13.13 region: seq[GRCh38] dup(16)(p13.13) chr16:g.11263062_12513616dup(Fig. 2). A total of 101 genes were deleted in the affected region. Additionally, 19 genes were identified in the duplicated region.

**Fig. 3:**
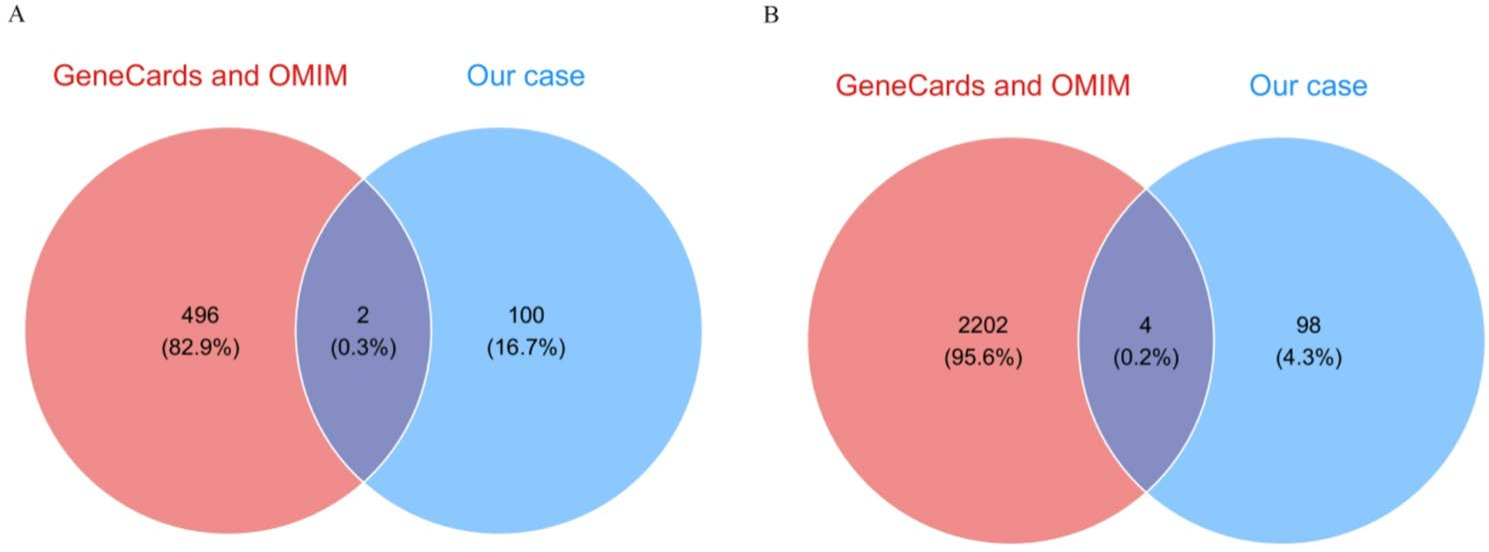
Venn diagrams showing the overlap between related genes from databases and deleted genes in the present case. (**A**) neurodevelopment-related genes from GeneCards and OMIM databases with duplicate entries removed were overlapped with the deleted genes identified in our case. (**B**) ovarian dysfunction-related genes from GeneCards and OMIM databases after excluding duplicateswere overlapped with the deleted genes detected in our case

A total of 498 genes related to neurodevelopment and 2206 genes related to ovarian dysfunction were collected from the GeneCards and OMIM databases after combining and deleting repetitive genes. The intersection between the genes related to neurodevelopment and ovarian dysfunction and the deleted genes in our case was then identified, serving as potential genes for causal genes. Ultimately, 2 overlapping genes, including *WFS1 and DRD5*, for the neurodevelopment (Fig. 3A) and 4 ovarian dysfunction-related genes(Fig. 3B), including *WFS1*,* CC2D2A*,* PROM1 and QDPR*, were identified. A review of the literature revealed 37 (Supplementary Table 1) [12–30] reported cases of proximal 4p deletion syndrome, with 16 cases (Table 1) exhibiting an overlap with the deleted region in our patient’s case.

**Table 1:** Phenotypic Features of 4p Interstitial Deletion Syndrome (Excluding 4p16.3) and Overlapping Deletion Regions: Comparison with Our Patient

	Our case	Romain et al., 1985 [15]	Davies et al., 1990 [16]	Chitayat et al., 1995 [19]		White et al.,1995 [21]			Innes, A. M et al.,1999 [23]	Tonk et al.,2003 [24]		Bailey et al., 2010 [4]	Li, D.et al.,2022 [10]	Pang, Y.et al.,2024 [5]	Total
Deleted region	4p15.32-16.2.2.2	4p15.2–15.33.33	4p15.2–16.1	4p15.2–15.33.33	4p15.2–15.33.33	4p14-16.1(NO.1)	4p15.2–16.1.1(NO.2)	4p15.1–16.1.1(NO.3–6)	4p15.2–16.1.1	4p15.2–16.1.1	4p15.2–16.1.1	4p14-15.33	4p15.32-16.1.1.1	4p14-15.33	17
Age	20y	32y	15.6y	10y	11y	14y	5m	10y	3y	35y	6y	2y	27m	7m	
Sex	F	F	M	M	F	M	F	F	F	F	F	F	F	M	
Long face	-	-	-	+	+	+	+	-	-	+	+	-	-	-	6/17
Upslanted fissures	-	-	-	-	+	+	+	+	-	-	-	+	+	-	9/17
Epicanthal folds	-	-	-	-	-	+	+	+	-	+	+	+	-	-	9/17
Large beaked nose	-	-	-	+	+	+	-	+	-	+	+	+	-	+	11/17
High or cleft palate	-	-	-	+	+	+	+	+	-	-	-	-	-	-	8/17
Thick lower lip	-	-	-	+	-	+	+	+	+	+	+	-	-	-	10/17
Micrognathia	-	-	-	-	-	+	+	+	-	-	-	-	-	-	6/17
Broad/short neck	-	-	-	+	+	+	+	-	-	-	-	-	-	-	4/17
Broad hands/feet	-	-	-	+	-	+	+	+	+	-	-	-	-	-	8/17
Tall, thin habitus	+	n/r	+	+	+	+	+	+	-	+	+	-	-	-	12/17
Mental retardation	+	+	+	+	+	+	+	+	+	+	+	+	+	+	17/17
Hypotonia	-	+	-	-	+	+	+	+	-	-	-	+	+	+	10/17
Epilepsy	-	-	-	-	-	-	-	-	-	-	-	-	-	-	0/17
Congenital heart disease	-	-	-	-	n/r	+	+	-	-	-	-	+	-	+	4/17
Cryptorchidism	-	n/r	-	+	n/r	+	-	-	-	-	-	n/r	-	-	2/17

Studies are listed by the name of the first author. M: male; F: female; n/r: not reported; y: years; mo: months; nb: newborn; +: present; –: absent

## Materials and methods

### Cytogenetic analysis

Peripheral blood was cultured and harvested using standard cytogenetic protocols. Chromosomal preparations were made using the G-banding technique, which has a resolution between 300 and 400 bands. Twenty metaphase cells were analyzed to determine the chromosomal karyotypes, following the guidelines of the International System of Human Cytogenetic Nomenclature (ISCN2024).

### NGS-CNV analysis

In addition, NGS-CNV was performed on the proband’s genomic DNA, which was extracted from PBMCs. This analysis was carried out using the NovaSeq 6000 platform from Illumina, based in San Diego, CA, United States.

### Acquisition of related genes

Neurodevelopment-related genes and ovarian dysfunction related genes were downloaded from databases such as Genecards and OMIM. A Venn diagram was generated using the R 4.4.2 software with R package(ggvenn).

## Discussion

This study describes a first Chinese patient with an 11.7 Mb deletion in 4p15.32-p16.2 and a 1.25 Mb duplication in 16p13.13, presenting with ovarian dysfunction, moderate intellectual disability and tall and thin habitus. Classical proximal 4p deletion syndrome, typically caused by deletions involving the 4p16.1-p14 region, is characterized by mild-to-moderate intellectual disability, subtle craniofacial dysmorphisms (e.g., long face, up-slanting palpebral fissures), and skeletal abnormalities (e.g., pectus excavatum, joint hyperlaxity) [28, 29]. Notably, the critical region underlying these classic phenotypes has been suggested to 4p15.2–15.33: a literature review of 19 cases demonstrated that larger deletions extending into 4p15.2–15.33 more frequently result in craniofacial dysmorphisms and skeletal abnormalities, presumably due to the disruption of a broader repertoire of genes [6, 10].

In contrast, our patient presented with only moderate intellectual disability without the aforementioned classic features; instead, she exhibited ovarian dysfunction, a manifestation not previously linked to proximal 4p deletions. This phenotypic discrepancy is attributable to the unique deletion interval in our case: a relatively small (11.7 Mb) deletion restricted to 4p15.32-p16.2, which spares most of the 4p15.2–15.33 critical region. Preservation of this critical region likely retained key genes governing craniofacial and skeletal development, thereby avoiding dysmorphic features. Importantly, the deleted interval still encompasses genes associated with cognitive function, which likely underpin the observed moderate intellectual disability.​Collectively, these observations highlight that the phenotypic spectrum of proximal 4p deletion syndrome is shaped by both the size of the deletion and the specificity of the involved interval—with disruption of the 4p15.2–15.33 critical region being central to the manifestation of classic craniofacial and skeletal abnormalities.

Through integration of database analysis (GeneCards, OMIM), we identified several candidate genes within the 4p deletion interval that may drive the observed phenotypes. The patient’s moderate intellectual disability (Level 3) aligns with the syndrome’s core feature. Among the deleted genes, DRD5 and WFS1 are potential candidates. DRD5, highly expressed in the prefrontal cortex, regulates cognitive function and memory, with its deletion linked to impaired neurodevelopment in murine models [31]. Regarding WFS1, while the 2012 ClinGen review (https://search.clinicalgenome.org/kb/gene-dosage/HGNC:12762) noted no evidence for haploinsufficiency and its classic association is with autosomal recessive Wolfram syndrome, recent findings [32] indicate heterozygous variants may contribute to neurological phenotypes, potentially via partial loss of function. In the context of this 4p deletion, reduced WFS1 expression could, alongside DRD5 haploinsufficiency, contribute to the patient’s intellectual disability.

Our study identifies ovarian dysfunction as a novel phenotypic feature associated with proximal 4p deletions, expanding the clinical spectrum of this chromosomal disorder. This finding contrasts with the report by Tonk et al., who described familial interstitial deletions of 4p15.2-p16.1 without observing reproductive impairment in affected individuals [24]. We hypothesize that this phenotypic discrepancy may stem from differences in the deleted chromosomal segments: whereas the deletion in the Tonk cohort was limited to 4p15.2-p16.1, our case involves a more extensive deletion spanning 4p15.32-p16.2, which may encompass additional genes critical for ovarian function. Within the deleted region, four candidate genes (*WFS1*, *CC2D2A*, *PROM1*, and *QDPR*) warrant detailed discussion regarding their potential roles in ovarian dysfunction. Among the four candidate genes identified (*WFS1*, CC2D2A, *PROM1*, *QDPR*), *WFS1* and *PROM1* have plausible roles in ovarian function. In addition to its critical role in neurodevelopment, *WFS1* is expressed in ovarian granulosa cells, and its mutations are associated with premature ovarian insufficiency (POI) in humans [33, 34]. *PROM1* (prominin 1) encodes a stem cell marker critical for folliculogenesis; murine models with Prom1 deletion exhibit impaired ovarian follicle maturation [35]. While *CC2D2A* and *QDPR* are primarily linked to ciliopathies and neurotransmitter metabolism, respectively [36, 37], their potential roles in ovarian biology warrant exploration, as ciliary dysfunction is increasingly recognized as a driver of POI [38].

We also identified a 1.25 Mb duplication at 16p13.13 encompassing 19 OMIM-annotated genes. Although the exact clinical significance of this specific 16p13.13 microduplication remains to be fully established, the wider 16p13.3-p13.1 region is now recognised as a recurrent CNV hotspot. Microduplications and microdeletions within this interval have been associated with variable neurodevelopmental and dysmorphic phenotypes, including epilepsy, learning difficulties and facial dysmorphism [39]. Similarly, 16p13.11 microdeletions and de novo 16p13.13 deletions [40] have been linked to global developmental delay and language impairment. These observations support the possibility that dosage-sensitive genes within 16p13.13 may contribute—either additively or synergistically—to the neurological features observed in our patient. Parental chromosomal analysis was offered but declined; therefore inheritance pattern could not be determined.

In summary, this report describes the first Chinese case of proximal 4p deletion syndrome, characterized by a unique phenotypic profile of ovarian dysfunction, and moderate intellectual disability. Our findings expand the clinical and genetic spectrum of the disorder and implicate *WFS1*, *DRD5*, *CC2D2A*, *PROM1*, and *QDPR* as candidate genes. These insights may improve clinical recognition of proximal 4p deletion syndrome, particularly in understudied populations, and advance efforts to identify its molecular basis.

## Supplementary Information

Supplementary Material 1.

## Data Availability

The data that support the findings of this study are available on request from the corresponding author.
